# Encoding of information using neural fingerprints

**DOI:** 10.1186/1471-2202-16-S1-P142

**Published:** 2015-12-18

**Authors:** José Luis Carrillo-Medina, Roberto Latorre

**Affiliations:** 1 Departamento de Eléctrica y Electrónica, Universidad de las Fuerzas Armadas ESPE, Sangolqui, Ecuador; 2 Grupo de Neurocomputación Biológica, Dpto. de Ingeniería Informática, Escuela Politécnica Superior, Universidad Autónoma de Madrid, 28049 Madrid, Spain

## 

The existence of *neural fingerprints *associated to specific cell types or to different processing states has been reported in widely different neural systems (e.g. see [1-3]). Does the nervous system have the ability to process information using these neural fingerprints? Model simulations suggest that *neural signatures *characterizing the origin of specific signals may play an important role for the fast and fine tuning of CPG rhythms [4], being a possible mechanism to *contextualize *or *discriminate *neural information in these circuits [5]. However, neural fingerprints can be a mechanism to encode temporal information in other neural networks of the nervous system.

In this work we investigate the ability of a simple network model to implement a neural processing based on the emission and recognition of neural fingerprints. We build two-dimensional networks of 50x50 binary neurons that communicate by exchange of serial binary patterns. Each unit is connected to eight other neurons using different network topologies. In particular, we study the influence of small-world topologies with different rewiring probability - ranging from regular to random networks - on the self-organizing properties of the network. In addition to the eight connection channels, another channel is used to introduce external stimuli in the network. Each individual neuron has the ability to recognize different neural fingerprints arriving thought its input channels in the form of specific binary patterns, i.e. in the model a fingerprint consists of a specific sequence of bits. When a fingerprint is recognized in the input, the neuron emits the same fingerprint to all its neighbors with probability p_r_. If no signature is recognized in a time step, the neuron emits a spontaneous pattern with probability p_e _(p_e _<< p_r _). After emitting a pattern, neurons have a refractory period during which neither emission nor recognition are made.

The proposed simple network is able to generate complex collective dynamics [6]. Without stimuli, the network evolves to a stationary state, related to the emission of the spontaneous activity. This does not depend on the network topology. When external stimuli are injected into the network (both in series and in parallel), the different neural fingerprints received through the external channels can propagate throughout the whole ensemble and the collective dynamics drastically changes. In this situation, localized patterns of activity related to the travelling neural fingerprint arise in the network. The intraunit parameters and the organization of connections between neurons tune the self-organizing properties within the network and define the spatial organization of the coexisting spatio-temporal patterns encoding the different stimuli. These patterns can survive and compete for long periods after the end of the stimulation, providing both short-term and long-term memory mechanisms to the network. Random connections enhance the fingerprint processing and potentiate long-term memory mechanisms. Conversely, regular patterns of connectivity promote short-term memory mechanisms and increase the encoding/storage capacity. These results point out that neural systems could process and encode information using neural fingerprints.
